# A physical mechanism of cancer heterogeneity

**DOI:** 10.1038/srep20679

**Published:** 2016-02-08

**Authors:** Cong Chen, Jin Wang

**Affiliations:** 1 Physics Department, Stony Brook University, NY 11794; 2 Chemistry Department, Stony Brook University, NY 11794.; 3 State Key Laboratory of Electroanalytical Chemistry, Changchun Institute of Applied Chemistry, Chinese Academy of Sciences, Changchun, Jilin 130022.

## Abstract

We studied a core cancer gene regulatory network motif to uncover possible source of cancer heterogeneity from epigenetic sources. When the time scale of the protein regulation to the gene is faster compared to the protein synthesis and degradation (adiabatic regime), normal state, cancer state and an intermediate premalignant state emerge. Due to the epigenetics such as DNA methylation and histone remodification, the time scale of the protein regulation to the gene can be slower or comparable to the protein synthesis and degradation (non-adiabatic regime). In this case, many more states emerge as possible phenotype alternations. This gives the origin of the heterogeneity. The cancer heterogeneity is reflected from the emergence of more phenotypic states, larger protein concentration fluctuations, wider kinetic distributions and multiplicity of kinetic paths from normal to cancer state, higher energy cost per gene switching, and weaker stability.

Tumor cells are known to have remarkable variability in phenotypes, a phenomenon known as heterogeneity. The distinct phenotypes lead to diversified biological behaviour. This is important in cancer research and clinical therapy. The origin of heterogeneity is closely related to the mechanism of cancer^1^. The idea of Darwinian-like clonal evolution is based on the discovery of acquisition of oncogenic mutations. It stresses the gene-centric development in which heterogeneity arises from the diversity of genotypes resulting from clonal evolution. On the other hand, the cancer cells are often hierarchically organized into nontumorigenic and tumorigenic cells with distinct phenotype manifestations^2^,^3^. The differences in tumorigenic potential within the same tumor is largely determined by epigenetic diversification. In other words, the origin of heterogeneity is often non-genetic^4^. The heterogeneity can be from the epigenetics and micro-environment such as DNA methylation and histone modifications. It can also come from stochastic nature of biological and chemical reactions involved. More and more evidences show that genetic heterogeneity is not likely to make major contribution to cancer heterogeneity^5^,^6^,^7^,^8^. Epigenetic origin of cancer heterogeneity is of both academic and clinical interest.

The cancer was often thought to be determined by the individual gene mutations. More evidences accumulated that cancer is a disease state emerged from the whole gene network, rather than only through individual gene mutations^9^,^10^. The results of dynamics and evolution for the gene network are largely determined by the topology of the network while at the same time the gene network dynamics is stochastic with intrinsic fluctuation from statistical molecular number fluctuations and external fluctuation from cell environment^11^,^12^. The deterministic and stochastic dynamics of cancer core network can provide possible source for cancer heterogeneity.

In cancer gene regulatory network, a key motif often emerged for function and cell fate decision making is the self activation and mutual repression^13^,^14^,^15^. For instance Rac1/RhoA circuits mediates amoeboid/mesenchymal transitions in Metastatic carcinoma cells^16^. miR200/ZEB double negative circuits in many cancer cells^17^. To explore heterogeneity arising from network dynamics, we will study one such cancer gene motif.

The key character of the dynamics for this cancer gene motif is the involvement of multiple timescales: timescale of protein synthesis/degradation and timescale of gene regulation or gene state switching processes. Adiabaticity is introduced to quantify the hierarchy of these time scales^15^,^18^,^19^,^20^,^21^,^22^. Many studies are concentrated in adiabatic limit where the rates for gene state switching due to regulations are much faster than the rates of protein synthesis/degradation. At this limit, adiabatic approximation is valid and driving force often has the form of Hill function^19^,^23^. Two major stable states often emerge in this limit with one gene activated and the other repressed. We identify these two states to be normal state and cancer state. Analytical and numerical studies show that under certain conditions there can be a third intermediate state in addition to normal and cancer states. The intermediate state has the nature of pre-malignant state^14^,^24^,^25^,^26^,^27^.

Although adibatic (fast regulation) assumption might be good for some prokaryotic cells such as bacteria, the regulation time scales can be elongated by the epigenetic factors in eukaryotic cells such as DNA methylation and histone remodifications. Slow regulatory binding has been shown to be important for cell fate decision making and stem cell differentiation and development^18^,^19^,^20^,^21^,^28^,^29^. As has been pointed out, though histone modification of single histones can be quick and frequent, the cooperative change of many histones which represents histone states occurs on a much slower time scale accompanied by dynamical DNA methylation/demethylation^22^,^30^. When TF binding/unbinding and histone modifications are considered explicitly, one can introduce the corresponding “adiabaticity”. In other words it introduces two levels of timescale hierarchy. The timescale hierarchy between transcription/degradation and regulation processes, and timescale hierarchy within regulation processes. For our model we only consider TF binding/unbinding as the regulation mechanism for simlicity. Thus our model uses one time scale to represent the complexity of the time scale hierarchy. Since dynamical effects from epigenetics (histone modification and DNA methylation) are reflected from their corresponding timescales. We use the regulation timescale to effectively represent the whole timescale related to epigenetics and regulation. We believe our simplified model provides a reasonable approximation and captures the essential feature of epigenetics. When the reaction time scale is extended to non-adiabatic regime with slower regulation. The translation/transcription and degradation processes happen more frequently compared to the regulatory processes. The gene states rarely change and there’s sufficient time for protein copy number to reach transcription level determined by the gene state. This usually leads to slower epigenetic states change reflected from their corresponding timescales.

In this study we suggest a possible source of cancer heterogeneity as being from epigenetics through gene regulation dynamics in the non-adiabatic (slow regulation) regime. The cancer heterogeneity is reflected from the emergence of more phenotypic states, larger protein concentration fluctuations, wider distribution of kinetics and multiplicity of paths from normal to cancer state, higher energy cost per gene switching and weaker stability.

## Model

**Figure 1:**
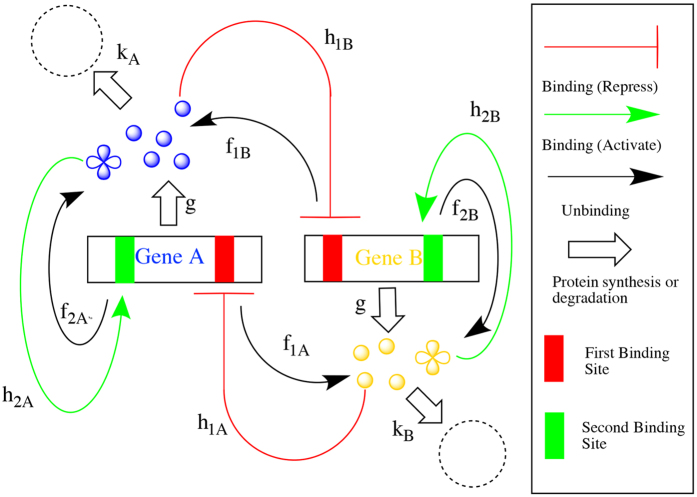
Regulation scheme of self activating mutually repressing regulatory motif.

We choose our cancer gene regulatory motif with mutually repression and self activation through transcription factor binding/unbinding. Figure 1 shows the regulation scheme of the two genes. Gene A and gene B each has 2 binding sites. The first binding site can be bound to a monomer produced by the other gene and the synthesis rate will be suppressed by a factor *λ*_*R*_ at the bound state (representing the repression gene regulation). The second binding site can be bound to a tetramer produced by itself and the synthesis rate will be raised by a factor *λ*_*A*_ (representing activation gene regulation). In previous similar gene regulatory network motif models, the form of transcription factor regulation is usually chosen from monomer to tetramer, such as monomer-tetramer and dimer-dimer, etc.^13^,^20^,^27^. While there is no direct experiment evidence as of which form transcription factor takes, we make a reasonable assumption that TF factors take monomer-tetramer form to represent the coorperativity of the regulatory binding consistent with previous studies. The 4 discrete states of each gene has protein synthesis rates set as: *g*_00_, *g*_01_ = *g*_00_*λ*_*A*_, *g*_10_ = *g*_00_*λ*_*R*_, *g*_11_*λ*_*A*_*λ*_*R*_. The first index *i* in *g*_*ij*_ represents the first binding site being at bound (1) state or unbound (0) state, the second index *j* represents the second binding site. The degradation rate for both proteins is set as *k* = 1. For simplicity, the unbinding rate for all binding sites is set as *f*. The binding rate for the first binding site (of both gene A and B) is given as 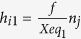 and for the second binding site is given as 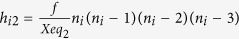. *Xeq*_1_ and *Xeq*_2_ are equilibrium constants(ratio of binding and unbinding kinetics). The adiabatic parameter is defined as *ω* = *f*/*k*.

Conventional studies for gene regulation dynamics are concentrated at adiabatic limit of fast regulation binding/unbinding compared to the protein synthesis/degradation. In this case, adiabatic approximation is valid and underlying stochastic dynamics can be described by the dynamic master equation, which has the form of 2-dimensional Fokker-Planck equation in the continuous limit^19^. Deterministic part of the driving force has the form of Hill function^14^ while the stochastic part of the force can come from the intrinsic statistical number fluctuations in concentrations or external fluctuations. The problem is greatly simplified. In the more general case, we should use master equation explicitly to describe the stochastic dynamics. Since each gene has four discrete states (00, 01, 10 and 11). The master equation which governs the dynamics, is 2^4^ = 16 dimensional. At large volume limit, the protein concentration variables *x*_*i*_ = *n*_*i*_/*V* become continuous. The master equation becomes ‘Coupled Fokker-Planck’ equation. It has 16 discrete ‘Fokker-Planck’ states that correspond to the system being at one of the 16 gene states. These Fokker-Planck states are coupled by the binding/unbinding reactions. There are two crucial time scales involved in such system: the timescale of protein synthesis/degradation and the timescale of binding/unbinding of regulatory gene network. Adiabatic parameter *ω* = *f*/*k* as the ratio between protein regulation binding/unbinding rate to the gene and the protein synthesis/degradation rate is introduced to quantify the hierarchy of the two timescales. The probability evolution follows the equation:


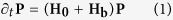


In the above coupled Fokker-Planck equation (master equation in large volume limit), the population or probability **P** is a 16 component state vector. Each component *P*_*s*_ stands for the probability of the system with protein concentration **x** being at gene state ‘s’. **H**_**0**_ and **H**_**b**_ are operators that can act on **P**. **H**_**0**_ is diagonal with 16 operators that describe the protein synthesis and degradation processes. **H**_*b*_ is non-diagonal with binding/unbinding terms that describe the ‘coupling’ between the gene states. The physical picture of coupled system is clear. Each component of **H**_**0**_ defines a probability landscape corresponding to a discrete state of the four binding sites being at a specific bound/unbound gene state. **H**_**b**_ describes the ‘hopping’ processes between these states. The adiabatic parameter *ω* is a measure of the relative strength of regulatory binding/unbinding of protein to the gene and protein synthesis and degrdation. When *ω* is large, **H**_**b**_ dominates over **H**_**0**_, the ‘hopping’ happens so frequently that the ‘hopping’ processes reaches an equilibrium and adiabatic regime of fast regulation is reached. When *ω* is small, the ‘hopping’ is rare and the system tends to stay in one of the 16 gene state landscapes. The non-adiabatic regime of slow regulation is reached. The moderate *ω* non-adiabatic regime is of special interest where proper approximation or analytical treatment is often lacking.

In practice, we use Gillespie algorithm^31^ to solve the coupled Fokker-Planck equation (master equation) from which we obtain the steady state probability that gives the underlying landscape of the network system quantifying the probability of the emergent phenotypic states and the evolution dynamics in time covering all the regimes of interests from adiabatic to non-adiabatic case and in between. Various analytical approximations made in adiabatic and non-adiabatic regimes are consistent with the full Gillespie simulations. We also developed a method for quantifying the optimal path in both adiabatic and non-adiabatic regime. Details are included in the supporting information.

## Results and Discussions

### Cancer Heterogeneity from Landscape View

In our model, there are two time scales quantifying the interaction strengths of the gene networks. One is the protein synthesis (g) and degradation rates (k), while the other is the binding(h)/unbinding(f) of regulatory proteins to the gene leading the gene state to switch. When the relative time scale *ω* = *f*/*k* is small, then the regulation processes are relatively slow and the gene switch (on and off) slower than the protein synthesis and degradation. In this non-adiabatic regime, the couplings among genes through protein regulations are loose due to the weak regulations. The individual genes can be either switched on or off without much influences from others. In this non-adibatic regime, the number of states one expects from the gene network can reach up to 2^*N*^ where N is the number of genes. When the relative time scale *ω* = *f*/*k* is large, then the regulation processes are fast and the genes switch faster than the protein synthesis and degradation. In this adiabatic regime, the couplings among genes are tight due to the strong regulations. The on and off states of genes are controlled by the interactions with other genes. Therefore, one expects that only finite number of states emerge as a result of gene interactions.

Due to the intrinsic and extrinsic fluctuations, the gene network dynamics is stochastic. Following the individual stochastic trajectory will not provide global information. We explore the probability evolution instead. The steady state probability landscape quantifies the chances of each individual state. It gives a global description for the network system. From the simulations of the kinetic processes involved in our cancer gene network motif, we see the steady state probability (*P*_*ss*_) and potential landscape (*U* = −*lnP*_*ss*_) projected in two and three dimensions with respect to two protein concentrations (gene products). Heterogeneity is directly related to the number of attractors of the landscape. The numbers and locations of the attractors determine the possible phenotypes that can be observed^25^,^32^,^33^.

**Figure 2:**
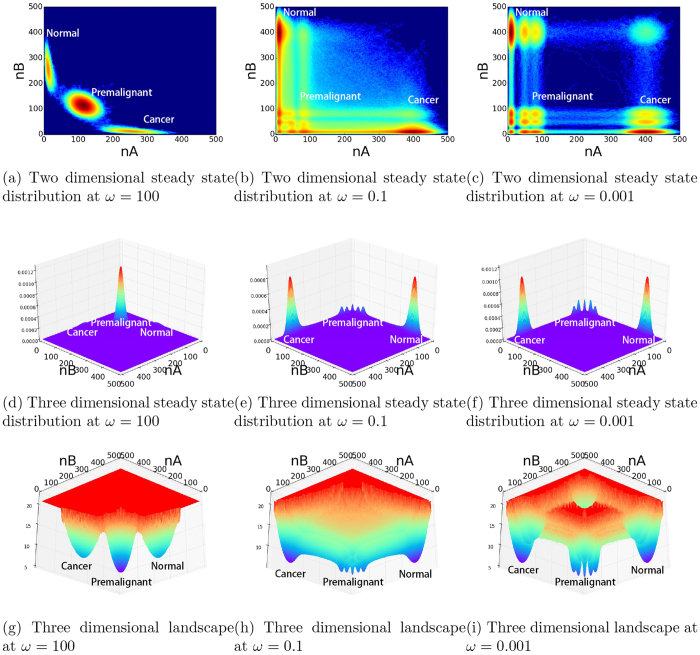
Two and Three dimensional landscape from fast regulation adiabaticity to slow regulation non-adiabaticity at *ω* = 100, 0.1, 0.001. (**a**) Two dimensional steady state distribution at *ω* = 100. (**b**) Two dimensional steady state distribution at *ω* = 0.1. (**c**) Two dimensional steady state distribution at *ω* = 0.001. (**d**) Three dimensional steady state distribution at *ω* = 100. (**e**) Three dimensional steady state distribution at *ω* = 0.1. (**f**) Three dimensional steady state distribution at *ω* = 0.001. (**g**) Three dimensional landscape at *ω* = 100. (**h**) Three dimensional landscape at *ω* = 0.1. (**i**) Three dimensional landscape at *ω* = 0.001.

As shown in Fig. 2(a,d,g), in the adiabatic regime when the gene network is strongly coupled (gene regulation time scale is much faster than the protein synthesis and degradation), three states emerge. Two states are mutually repressive to each other. One state has one gene on and the other gene off. This leads to the state with one gene expression high and the other gene expression low. Here gene expression is represented by the corresponding protein concentration. The other state is just the opposite. The high expression of the first gene and low expression of the second gene can be used to represent the cancer state, while the high expression of the second gene and low expression of the first gene can be used to represent the normal state. Then we see both normal and cancer states emerge from the gene network motif. Furthermore, both states are quantified by the two basins of attractions on the landscape with large probability. The reason of the normal and cancer state/basin appearance lies in the fact of the mutual repressing interactions among the genes. We also notice that a third state quantified by the basin of attraction emerges representing an gene on-gene on state. The appearance of this state/basin is from the self activation of both genes. Both experimentally and clinically, premalignant states between normal and cancer have been observed^34^,^35^. The emergence of intermediate state/basin between normal and cancer state in this core cancer gene regulatory motif may shed lights on quantifying the premalignant state.

As shown in Fig. 2(b,c,e,f,h,i), in the non-adiabatic regime when the gene network is weakly coupled (gene regulation time scale is slower than the protein synthesis and degradation), more states quantified by local basins of attractions emerge. As mentioned, the slow regulations can reflect epigenetic effects of extra time scales from DNA methylation and histone regulations. The normal, intermediate premalignant and cancer state basins still persist under slow regulation regime. Besides these three major state basins (normal, cancer and intermediate state), we have observed the emergence of many local state basins around the major basins. The multiple local basins around the major basins give a quantitative picture of the cancer heterogeneity.

We also see the emergence of other states. Cancer and premaliganant states are not individual states but composed of many states as we can see clearly under the epigenetic conditions (relatively slow regulations). The possible physical mechanism of the cancer heterogeneity is thus from the weakening of the regulatory interactions among genes in the gene network. The epigentics such as DNA mythylation and histone remodification can naturally lead to the effective weakening of the gene interactions. The results of our model show that both premalignant intermediate and heterogeneity can coexist under epigenetic conditions. This is consistent with the experimental findings^36^. Our model gives the underlying physical mechanisms behind this. The regulatory interactions lead to the premalignant intermediate state (the self activation in this network motif) while the epigenetic interactions lead to cancer heterogeneity.

From another angle, we can see as the gene network is weakly coupled due to the weakening of interactions in **H**_*b*_ of Eq. (1). (with epigenetics of DNA methylation and histone remodification being possible source), one expects to have the emergence of many states or phenotypes. The state basins are relatively shallow. Thus this is a possible physical mechanism of cancer heterogeneity. As the coupling among genes become stronger, more and more states are merged together. Shallower basins are merged to deeper and larger basins. As a result, there are less phenotypic states but relatively more stable. While the weak couplings between genes (or landscapes of fixed gene states) at non-adiabatic regime can explain qualitatively the emergence of alternative phenotypic states, we will perform a quantitative study of fluctuation, stability, state transitions and thermodynamic properties to further uncover the relationship between heterogeneity and time scale (non-adiabaticity) of the regulation dynamics. The epigenetics can be seen as environments of genes. The effects or strengths of epigenetics physically can be quantified through the dynamical timescale involved.

The heterogeneity in terms of concentrations, fluctuations through Fano factors and associated distributions on landscape topography shows similar heterogeneity from intermediate to extreme non-adiabaticity (since intermediate non-adiabaticity we chose here already represents enough weak couplings that almost all possible phenotypic states emerge). The above similarity in heterogeneity can be further differentiated through the kinetics. This is done through the explorations in quantitative details of heterogeneity from kinetic rate and path transition perspectives in supporting information. We see that intermediate level of non-adiabaticity seems to give more kinetic heterogeneity due to the interplay of regulation and synthesis/degradation time scales.

### Cancer Heterogeneity from Fluctuations in Concentrations and Kinetics

**Figure 3:**
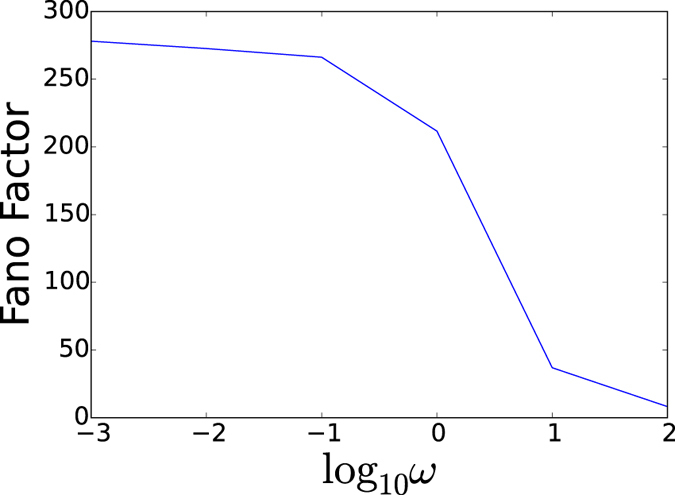
Fano factor from slow regulation non-adiabaticity to fast regulation adiabaticity.

We can further characterize the cancer heterogeneity from the fluctuations in concentrations and kinetics. Figure 3 shows the Fano factor which is the ratio of variance versus the mean of the protein concentrations (gene products). In a pure random process, statistical fluctuations from intrinsic noise has a Poisson nature in which case Fano factor is equal to 1. We see when the adiabaticity parameter *ω* of relative regulatory binding/unbinding to protein synthesis and degradation is large, the fluctuations is non-Poisson in this adiabatic regime. This is due to the nature of underlying reactions of protein synthesis and degradation. On the other hand, when the adiabaticity parameter *ω* of relative regulatory binding/unbinding to protein synthesis and degradation is small, the fluctuations are significantly larger. The significant larger fluctuations give the variances and heterogeneity. This heterogeneity is coming from the emergence of the many local state basins of attractions.

**Figure 4:**
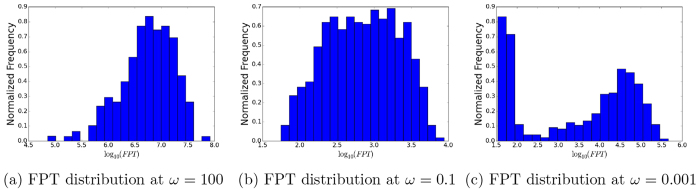
FPT distribution from slow regulation non-adiabaticity to fast regulation adiabaticity. (**a**) FPT distribution at *ω* = 100. (**b**) FPT distribution at *ω* = 0.1. (**c**) FPT distribution at *ω* = 0.001.

In Fig. 4(a–c), we also show the heterogeneity in kinetics. We plot the statistical distribution of first passage time from normal to cancer state. We see when the adiabaticity parameter *ω* of relative binding/unbinding to synthesis and degradation is large (Fig. 4(a)), the distribution of kinetics is rather narrow in this adiabatic regime. This is due to the limited kinetic paths between normal and cancer state basins. On the other hand, when the adiabaticity parameter *ω* of relative binding/unbinding to synthesis and degradation becomes smaller (Fig. 4(b)), the statistical distribution of the kinetics is significantly wider. The significant wider distribution indicates that there are many more pathways from normal to cancer state basins. This shows the kinetic variance and heterogeneity. Again, this kinetic heterogeneity is coming from the emergence of the many local state basins of attractions. These new basins of attractions lead to a rougher landscape and therefore multiple non-equivalent pathways from normal to cancer state. Finally, when the adiabaticity parameter *ω* of relative binding/unbinding to synthesis and degradation is extremely small (Fig. 4(c)), the statistical distribution of the kinetics becomes narrower. Under this condition, the rate limiting step is the switching speed of the genes rather than the protein synthesis or degradations. Due to the slowness of the switching speed caused by the slow regulation, single or limited switchings dominate the kinetics. Therefore, although there are more local basins, there are only limited explorations under extreme slow switchings and the kinetic heterogeneity becomes less.

### Cancer Heterogeneity from Kinetic Paths, Energy Cost and Stability

**Figure 5:**
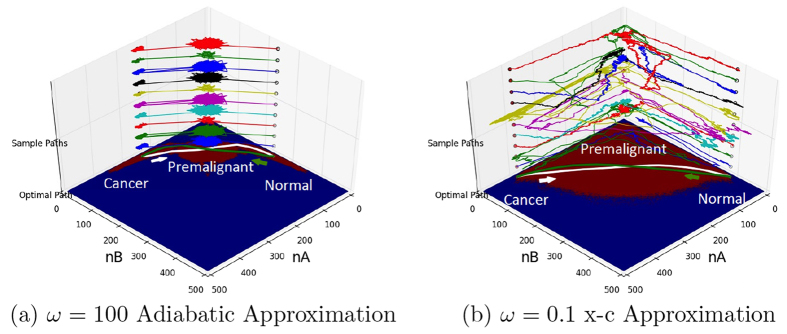
Compare optimal path(white and green) at adiabatic limit and moderate non-adiabatic region. Colored paths are real paths generated by Gillespie simulation. (**a**) *ω* = 100 Adiabatic Approximation. (**b**) *ω* = 0.1 x−c Approximation.

We can see the effects of heterogeneity on the kinetic paths from normal state to cancer state. We show the optimal kinetic paths when the adiabaticity parameter *ω* of relative binding/unbinding to synthesis and degradation is large (Fig. 5(a)). We can see there is almost a unique path from normal state to cancer state through the intermediate premalignant state. The fluctuations around the optimal paths are small and the optimal path tube is narrow. However, when the adiabaticity parameter *ω* of relative binding/unbinding to synthesis and degradation is small (Fig. 5(b)), the optimal path from normal state to cancer state deviates from the adiabatic optimal path. This is due to the emergence of more local state basins of attractions, leading to the shifts for the optimal paths. Furthermore, we see the fluctuations around this optimal path is relatively large compared with the adiabatic path, resulting a wider optimal path tube from normal to cancer state. Again, this is due to the presence of the multiple local state basins. Notice the discussion on the path fluctuations here in relation to the heterogeneity is consistent with the discussion on the kinetic heterogeneity (Fig. 4) where wider distribution of kinetics implies multiple paths from normal state to cancer state.

**Figure 6:**
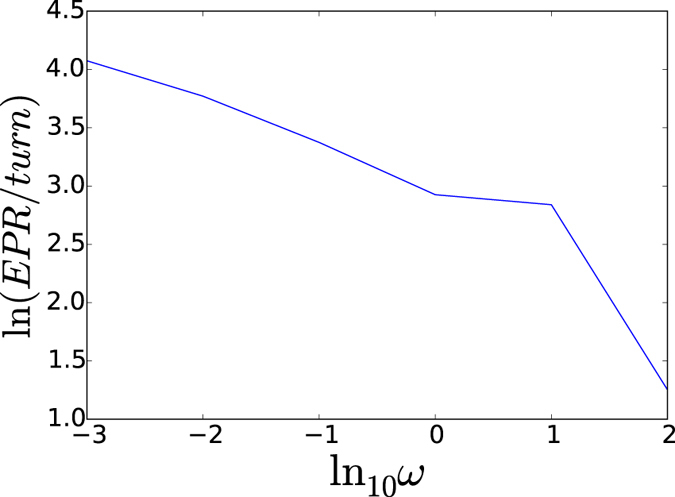
Energy cost per turnover from slow regulation non-adiabaticity to fast regulation adiabaticity.

The cancer heterogeneity can also be reflected by the energy cost per gene switching. Being a non-equilibrium dynamical system, there is energy or heat dissipation measured by the entropy production associated with the irreversibility from the non-equilibriumness^20^,^32^,^37^. Figure 6 shows the energy cost per gene switching from non-adiabaticity of slow regulation compared to protein synthesis/degradation to adiabaticity of fast regulation compared to protein synthesis/degradation. We see the energy cost per gene switching increases monotonically with respect to the slower regulation compared to the protein synthesis/degradation (decrease in *ω*). The increase of the energy cost is due to the heterogeneity in non-adiabatic slow regulation regime from the emergence of multiple state basins of attractions. Gene switchings cost more energy because of the number of state flipping is more.

**Figure 7:**
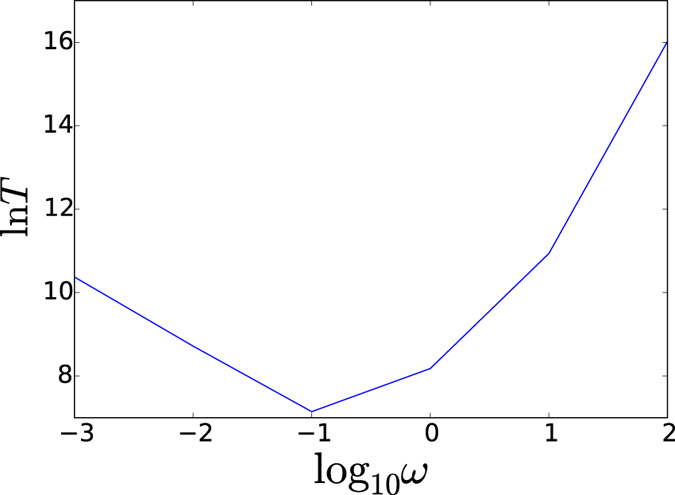
MFPT from slow regulation non-adiabaticity to fast regulation adiabaticity.

The heterogeneity can also be seen from the stability explorations. The stability of the normal and cancer state can be quantitatively measured by the kinetic time from one state to another. If the kinetic transition time from normal(cancer) state to cancer(normal) state is long, then the stability of normal(cancer) state is high. This is because the normal state can then be maintained for a long (short) time. In Fig. 7, we see the mean first passage time from normal to cancer state with respect to the adiabaticity parameter *ω* of gene regulation versus protein synthesis/degradation. The kinetics shows a non-monotonic behavior. At very low *ω* of extreme non-adiabatic regime with slow regulation, the rate limiting step is the gene switching. Increasing the regulation speed leads to the faster switching and therefore faster kinetics. On the other hand, as the regulation time scale becomes faster than the protein synthesis and degradation, there is no sufficient time for protein copy numbers to reach to the transcription level corresponding to the gene state for each gene switching. Therefore, the rate limiting step is no longer gene switching anymore. In fact, the adiabatic barrier from one transcription (concentration) level to another is determined by the protein synthesis/degradation averaged over the rapid gene switchings. The faster the regulation speed or gene switchings, the harder for the gene transcription in terms of protein concentration level to catch for the specific gene state, therefore the kinetics becomes longer. Therefore both faster and slower regulation or switching give slower kinetics, while in the moderate regime of *ω* of gene regulation or switching relative to protein synthesis/degration, there is an optimal kinetic speed from normal to cancer state. Therefore, from the kinetics, we can see the stability is high at large *ω* (adiabatic regime). This is due to the limited state basins with significant depths. For smaller *ω* (non-adiabatic regime), the faster kinetics emerges. This implies less stability. The lower stability is from the the emergence of the multiple states with shallower basins. The very small *ω* of extreme non-adiabatic regime only has a moderate increase of the kinetic time scale and therefore slightly higher stability relative to the optimal one, in contrast with the adiabatic case for much longer duration and higher stability. As we can see the lower stability is another reflection of the underlying heterogeneity.

## Conclusion

In this study, we use a core cancer gene regulatory motif to study the possible source of cancer heterogeneity. Normal state and cancer state, as well as intermediate states emerge as possible phenotype alternations in both adiabatic fast and non-adiabatic slow regulation regime. Slow regulations can come from epigentics of DNA methylation and histone remodification which lead to weaker coupling among genes. As a result, more steady states corresponding to more phenotype manifestations emerge. The cancer heterogeneity is reflected from the emergence of more phenotypic states, the larger transcription level concentration fluctuations, wider kinetic distributions and multiplicity of kinetic paths from normal to cancer state, more energy cost per gene switching, and weaker stability. The relationship between non-adiabatic slow regulation dynamics and epigenetic heterogeneity in cancer gene networks calls attention for further study.

## Additional Information

**How to cite this article**: Chen, C. and Wang, J. A physical mechanism of cancer heterogeneity. *Sci. Rep.* **6**, 20679; doi: 10.1038/srep20679 (2016).

## Supplementary Material

Supplementary Information
