# Supplementary material for: A physical mechanism of cancer heterogeneity
Source: Sci Rep. 2016 Feb 8;6:20679. doi: 10.1038/srep20679 (PMC4745067; doi:10.1038/srep20679)
Supplement: Supplementary Information [file srep20679-s1.pdf]

Supporting Information

A physical mechanism of cancer heterogeneity

Cong Chen[†] and Jin Wang[*,‡,†,¶]

†*Physics Department, Stony Brook University, NY 11794*

‡*Chemistry Department, Stony Brook University, NY 11794*

¶*State Key Laboratory of Electroanalytical Chemistry, Changchun Institute of Applied Chemistry, Chinese Academy of Sciences, Changchun, Jilin 130022*

E-mail: jin.wang.1@stonybrook.edu

Details of the network motif

For the self activating and mutually repressing network motif, the two genes Gene A and Gene B each has two binding sites. The first binding site of gene A(B) can be bound to a monomer produced by gene B(A) and the synthesis rate of protein A(B) will be repressed by a factor λ_R. The second binding site of gene A(B) can be bound to a tetramer produced by gene A(B) and the synthesis rate of A(B) will be raised by a factor λ_A. We are using multiplicative model with activation and repression effectively measured as multiplicative factors instead of conventional additive factors. We believe multiplicative model is more close in practice as to how gene regulatory network makes biological logic decisions. The synthesis rates for different bound/unbound states are $g_{00}, g_{10} = \lambda_R g_{00}$, $g_{01} = \lambda_A g_{00}$, $g_{11} = \lambda_R \lambda_A g_{00}$. The degradation rate for both protein A and protein B is set to be $k_A = k_B = k$. The unbinding rate for all the 4 binding sites are set equat $f_{1A} = f_{2A} = f_{1B} = f_{2B} = f = k \cdot \omega$. The binding rate for the first binding site of gene A(B) is $h_{1A} = \frac{f}{Xeq_1} n_B$ ($h_{1B} = \frac{f}{Xeq_1} n_A$). The binding rate for the second binding site of gene A(B) is $h_{2A} = \frac{f}{Xeq_2} n_A(n_A - 1)(n_A - 2)(n_A - 3)$ ($h_{2B} = \frac{f}{Xeq_2} n_B(n_B - 1)(n_B - 2)(n_B - 3)$). The model can be expressed by the chemical reactions:

$$\text{Gene}A^{0\alpha} + (n+1)B \underset{h_{1A}}{\overset{f_{1A}}{\leftrightarrows}} \text{Gene}_A^{1\alpha} + nB \qquad \text{Gene}A^{\alpha 0} + (n+4)A \underset{h_{2A}}{\overset{f_{2A}}{\leftrightarrows}} \text{Gene}A^{\alpha 1} + nA$$

$$\text{Gene}B^{0\alpha} + (n+1)A \underset{h_{1B}}{\overset{f_{1B}}{\leftrightarrows}} \text{Gene}_B^{1\alpha} + nA \qquad \text{Gene}B^{\alpha 0} + (n+4)B \underset{h_{2B}}{\overset{f_{2B}}{\leftrightarrows}} \text{Gene}B^{\alpha 1} + nB$$

$$nA \underset{g}{\overset{k}{\leftrightarrows}} (n+1)A \qquad nB \underset{g}{\overset{k}{\leftrightarrows}} (n+1)B$$

In above reactions, α represents an arbitrary state of the binding site, it can be 0 or 1. At large volume limit, protein concentration $x = \frac{n}{V}$ becomes continuous variable. For simplicity we absorb the volume 'V' into g, f and Xeqs. We set synthesis rates $g_{00} = 5$, $\lambda_A = 8$, $\lambda_R = 0.2$, degradation rate $k = 0.1$, unbinding rate $f = k \cdot \omega$ and binding rate $h_{1A} = \frac{f}{Xeq_1} x_B$ ($h_{1B} = \frac{f}{Xeq_1} x_A$), $h_{2A} = \frac{f}{Xeq_2} x_A^4$ ($h_{2B} = \frac{f}{Xeq_2} x_B^4$). Equilibrium constant for mutual repression

is set as $Xeq_1 = 15$ and for self activation $Xeq_2 = 50^4$. The master equation that governs network dynamics has the form of coupled Fokker-Planck equation:

$$\partial_t \mathbf{P} = (\mathbf{H_0} + \mathbf{H_b})\mathbf{P} \tag{1}$$

P is a 16 component vector whose component $P_s(x,t)$ represents the probability of the system being at gene state s with protein concentration x at time t.

$$\mathbf{P} = \begin{pmatrix} P_{1111}(x,t) \\ P_{1110}(x,t) \\ P_{1101}(x,t) \\ P_{1100}(x,t) \\ \vdots \\ P_{0000}(x,t) \end{pmatrix} \tag{2}$$

$\mathcal{H}_0$ describes protein synthesis and degradation processes. It is diagonal with 16 diagonal elements. Each describes a continuous landscape at corresponding discrete gene state.

$$\mathcal{H}_0 = \begin{pmatrix} \mathcal{L}_{1111} & 0 & 0 & 0 & \dots & \vdots \\ 0 & \mathcal{L}_{1110} & 0 & 0 & \dots & \vdots \\ 0 & 0 & \mathcal{L}_{1101} & 0 & \dots & \vdots \\ 0 & 0 & 0 & \mathcal{L}_{1100} & \dots & \vdots \\ \vdots & \vdots & \vdots & \vdots & \ddots & \vdots \\ \dots & \dots & \dots & \dots & \dots & \mathcal{L}_{0000} \end{pmatrix} \tag{3}$$

Each diagonal element $\mathcal{L}_{ijkl}$ (each index i, j, k, l can be either 0 or 1) is a 'Focker-Planck' operator:

$$\mathcal{L}_{ijkl} = -\partial_{x_A}(g_{ij} - k_A x_A) - \partial_{x_B}(g_{kl} - k_B x_B) + \frac{1}{2}\partial^2_{x_A}(g_{ij} + k_A x_A) + \frac{1}{2}\partial^2_{x_B}(g_{kl} + k_B x_B) \tag{4}$$

On the other hand, $\mathcal{H}_b$ describes binding/unbinding processes and is non-diagonal. It describes the 'coupling' between discrete gene states.

$$\mathcal{H}_b = \begin{pmatrix} r_{1111} & h_{2B} & h_{1B} & 0 & \dots & \vdots \\ f_{2B} & r_{1110} & 0 & h_{1B} & \dots & \vdots \\ f_{1B} & 0 & r_{1101} & h_{2B} & \dots & \vdots \\ 0 & f_{1B} & f_{2B} & r_{1100} & \dots & \vdots \\ \vdots & \vdots & \vdots & \vdots & \ddots & \vdots \\ \dots & \dots & \dots & \dots & \dots & r_{0000} \end{pmatrix} \tag{5}$$

The diagonal elements r_{ijkl} in $\mathcal{H}_B$ are escaping rates from gene state $ijkl$.

$$\begin{aligned} r_{1111} &= -f_{1A} - f_{2A} - f_{1B} - f_{2B} \\ r_{1110} &= -f_{1A} - f_{2A} - f_{1B} - h_{2B} \\ r_{1101} &= -f_{1A} - f_{2A} - h_{1B} - f_{2B} \\ r_{1100} &= -f_{1A} - f_{2A} - h_{1B} - h_{2B} \\ \vdots &= \vdots \quad \vdots \quad \vdots \quad \vdots \\ r_{0000} &= -h_{1A} - h_{2A} - h_{1B} - h_{2B} \end{aligned} \tag{6}$$

Adiabatic Limit from Analytical Approach

The gene network dynamics can be simulated with the Gillespie algorithm.[1] Various analytical approaches can be developed to approximate the adiabatic fast regulation and non-adiabatic slow regulation limit. When ω is large, we are at adiabatic limit where adiabatic approximation of fast regulation of protein to the gene compared to the protein synthesis/degradation is valid.[2,3]

When $\mathcal{H}_b$ terms representing binding/unbinding or gene switches dominate over H_0 terms representing protein synthesis/degradation, the dynamics of the fast degree of freedom (gene

switching) quickly reaches equilibrium which is the steady state of $\mathcal{H}_b$:

$$\mathbf{H}_b \xi = 0 \tag{7}$$

here ξ is a 16 components state vector and is normalized as $\mathbf{1}^T \cdot \xi = \sum_i \xi_i = 1$. Being close to ξ, the total probability distribution can be written as:

$$\mathbf{P} = \rho\xi + \epsilon \tag{8}$$

where ρ is a scaler function of x and t, ϵ is a small deviation from $\rho\xi$.

The probability of the gene network with x protein concentration is the sum over the probabilities of the network at x protein concentration but being at different gene states . That is $\mathbf{1}^T \cdot \mathbf{P} = \sum_i P_i \approx \rho$. Multiply the master equation using $\mathbf{1}^T$ from the left, and notice we always have $\mathbf{1}^T \mathbf{H}_b = 0$. The master equation becomes:

$$\partial_t \rho = \mathbf{1}^T H_0 \rho \xi = \sum_i (H_0)_{ii} (\xi_i \rho) \tag{9}$$

The right hand side is the sum of 16 Fokker-Planck equations result in the final form of 2-dimensional Fokker-Planck equation. The driving force has the form of Hill functions and intrinsic fluctuation is x dependent.

$$\frac{d}{dt}\rho = -\partial_{x_A}(F_A \rho) - \partial_{x_B}(F_B \rho) + \frac{1}{2}\partial_{x_A}^2 (D_A \rho) + \frac{1}{2}\partial_{x_B}^2 (D_B \rho) \tag{10}$$

with driving force and diffusion coefficients:

$$
\begin{aligned}
F_A = \quad & g_{00}\frac{f}{f+h_{1A}}\frac{f}{f+h_{2A}} + g_{01}\frac{f}{f+h_{1A}}\frac{h_{2A}}{h_{2A}+f} \\
& +g_{10}\frac{h_{1A}}{h_{1A}+f}\frac{f}{h_{2A}+f} + g_{11}\frac{h_{1A}}{h_{1A}+f}\frac{h_{2A}}{h_{2A}+f} - kx_A \\
F_B = \quad & g_{00}\frac{f}{f+h_{1B}}\frac{f}{f+h_{2B}} + g_{01}\frac{f}{f+h_{1B}}\frac{h_{2B}}{h_{2B}+f} \qquad (11) \\
& +g_{10}\frac{h_{1B}}{h_{1B}+f}\frac{f}{h_{2B}+f} + g_{11}\frac{h_{1B}}{h_{1B}+f}\frac{h_{2B}}{h_{2B}+f} - kx_B \\
D_A = \quad & g_{00}\frac{f}{f+h_{1A}}\frac{f}{f+h_{2A}} + g_{01}\frac{f}{f+h_{1A}}\frac{h_{2A}}{h_{2A}+f} \\
& +g_{10}\frac{h_{1A}}{h_{1A}+f}\frac{f}{h_{2A}+f} + g_{11}\frac{h_{1A}}{h_{1A}+f}\frac{h_{2A}}{h_{2A}+f} + kx_A \\
D_B = \quad & g_{00}\frac{f}{f+h_{1B}}\frac{f}{f+h_{2B}} + g_{01}\frac{f}{f+h_{1B}}\frac{h_{2B}}{h_{2B}+f} \qquad (12) \\
& +g_{10}\frac{h_{1B}}{h_{1B}+f}\frac{f}{h_{2B}+f} + g_{11}\frac{h_{1B}}{h_{1B}+f}\frac{h_{2B}}{h_{2B}+f} + kx_B \qquad (13)
\end{aligned}
$$

When the whole system reaches steady state (protein concentrations in addition to the gene switchings), we can quantify the landscape as :

$$U = -\ln \rho_{SS}(x) \qquad (14)$$

The stable states correspond to local minimum of the landscape U. The stability of the states is related to the topography of the landscape in terms of basin depths. The Fokker-Planck equation can also be written in terms of probability conservation with left hand side being the probability change in time and righthand side as the divergence of the flux. The physical meaning is clear. The probability increase and decrease is associated with the flux in and out. The flux is given as $j_i^{SS} = -F_i\rho^{ss} + \frac{1}{2}\partial_j(D_{ij}\rho^{ss})$ and is divergent free $\nabla \cdot \mathbf{j} = 0$ at steady state. Define $\hat{F}_i = F_i - \frac{1}{2}(\partial_j D_{ij})$, flux can be written as $\mathbf{j} = -\mathbf{F}\rho^{SS} + \frac{1}{2}\partial \cdot (\mathbf{D}\rho^{SS}) = -\hat{\mathbf{F}}\rho^{SS} + \frac{1}{2}\mathbf{D} \cdot \partial\rho^{SS}$, which is similar to constant diffusion case:. The driving force can be decomposed into a gradient part plus a curl part: $F_i = -\frac{1}{2}D_{ij}\partial_j U + j_i^{ss}/\rho^{ss} + \frac{1}{2}\partial_j D_{ij}$ or $\hat{F}_i = -\frac{1}{2}D_{ij}\partial_j U + j_i^{ss}/\rho^{ss}$. Non-zero flux is a measure of how far the system deviates from

the detailed balance (how far away the system is from equilibrium). The non-zero flux is the origin of the irreversibility and entropy production.[4,5]

As shown in Fig. 2 in the main text, simulations under adiabatic approximation confirms that besides normal state and cancer state, an intermediate state emerges as possible source of phenotype alternation. With the dynamic equation being 2-dimensional Fokker-Planck equation under adiabatic approximation, it is possible to quantify optimal path using least action approach. The 2D Fokker-Planck system is equivalent to a Lagrangian dynamic system with effective Lagrangian:[6,7]

$$\mathcal{L} = -\sum_{i,j} \frac{1}{2D_{ij}}(\dot{x}_i - F_i)(\dot{x}_j - F_j) + \sum_{i,j,k} \frac{1}{2} D_{ik} \partial_k (F_j D_{ij}^{-1}) \tag{15}$$

Path integral tells us that, among all the possible transition paths connecting initial (normal) state and final (cancer) state, the possibility of a single path is proportional to the exponential of the negative action $S = \int_{t_i}^{t_f} \mathcal{L} dt$. The optimal path is the one that minimizes action S.[8] As has been pointed out, by using kinetic equation and energy conservation along optimal path, the action can be simplified into a line integral along the path[9,10]

$$S = \int_{x_i}^{x_f} dl \sqrt{2(E - V_{eff}(x))} - \int_{l_i}^{l_f} dl \sum_{i,j} F_l \tag{16}$$

D_{ij}^{-1} plays the role of metric in the curved space. $dl = \sqrt{\sum_{i,j} D_{ij}^{-1} dx_i dx_j}$ is the 'distance'. F_l is the force projected to the path line modularized by the diffusion. The second term in the action suggests the path is irreversible since the force is not a pure gradient. The curl flux component of the force will lead to non-trivial contribution to the dynamics. This confirms the irreversibility of cancer model: the reversed optimal path will no longer be the optimal path as the new action is larger and the probability for the reversed path is smaller.

Fig. 5(a) shows clearly that at adiabatic limit, the optimal transition path going through the intermediate state. In other words, a large portion of the transition paths will go through intermediate state, resulting in a hub with portion of the cells showing the intermediate

alternative phenotype.

Non-Adiabatic Limit from Analytical Approach

As ω decreases, binding/unbinding processes are less frequent and we reach the non-adiabatic regime. Adiabatic approximation is no longer valid. We have to use 16 dimensional master equation explicitly.

In the small ω, or weak coupling limit. $\mathcal{H}_b$ coupling terms are much smaller than $\mathcal{H}_0$ terms. System tends to stay in one of the 16 states and rarely jumps to another. In this limit, besides normal state and cancer state, we are able to identify more intermediate states. Especially the 'off-off' state where both genes are repressed. These states have a great impact on the cancer transition behaviour. There are also smaller peaks emerging around the cancer state, suggesting possible phenotype alternations even after the system reaches cancer state.

The moderate ω non-adiabatic region is of special interest. Conventionally in this regima solid theoretical framework and analytical results are hard to achieve. Recent progress[6,11–13] showed that there's similarity between coupled Fokker-Planck equation and Schrodinger equation. One can represent Fokker-Planck equation in the operator form and introduce spinor representation to quantify discrete gene states. In other words, the coupled discrete (gene state) and continuous (protein degrees of freedom) processes are very hard to study analytically. However, we can map the problem to the total continuous representation so that analytical approach is possible. This is realized by monitoring the occupation or probability of the gene on and off states instead of the discrete labeling itself, since occupation is a continuous variable. The result is then the continuous processes in extended dimensions (new continuous variables including the occupations of the gene on and off states). Path integral can further map the Hamiltonian dynamics into Lagrangian dynamics with effective Lagrangian. After Hubbard-Stratonovich transformation the effective Lagrangian provides information of the dynamics at both classic (deterministic level) and semi-classical (intrinsic fluctuation) level in the extended continuous space.

In our case, there are 4 on/off binary binding sites, it is proper to introduce 4 two-components spinors. 4 auxiliary variables: $(c_{A0}, c_{A1}, c_{B0}, c_{B1})$ and their conjugate variables are introduced to quantify the dynamics of the spinors. Follow the path integral procedure with spinor representation, the original coupled Fokker-Planck system is mapped to a single Fokker-Planck equation in $2+4=6$ dimensional extended space.

$$
\begin{aligned}
\partial_t P = \quad & -\sum_{i=1}^{i=2} \partial_{x_i}(\tilde{F}_{x_i} P) - \sum_{j=1}^{j=4} \partial_{c_j}(\tilde{F}_{c_j} P) \\
& + \sum_{i=1}^{i=2} \frac{1}{2} \partial^2_{x_i}(\tilde{D}_{x_i} P) + \sum_{j=1}^{j=4} \frac{1}{2} \partial^2_{c_j}(\tilde{D}_{c_j} P)
\end{aligned}
$$

with driving force and diffusion coefficients:

$$
\begin{aligned}
\tilde{F}_{x_A} &= c_{A0} c_{A1} g_{00} + c_{A0}(1 - c_{A1}) g_{01} \\
& \quad +(1 - c_{A0}) c_{A1} g_{10} + (1 - c_{A0})(1 - c_{A1}) g_{11} - k \cdot x_A \\
\tilde{F}_{x_B} &= c_{B0} c_{B1} g_{00} + c_{B0}(1 - c_{B1}) g_{01} \\
& \quad +(1 - c_{B0}) c_{B1} g_{10} + (1 - c_{B0})(1 - c_{B1}) g_{11} - k \cdot x_B \\
\tilde{F}_{c_{A0}} &= f(1 - c_{A0}) - \frac{f}{Xeq_1} x_B c_{A0} \\
\tilde{F}_{c_{A1}} &= f(1 - c_{A1}) - \frac{f}{Xeq_2} x_A c_{A1} \\
\tilde{F}_{c_{B0}} &= f(1 - c_{B0}) - \frac{f}{Xeq_1} x_A c_{B0} \\
\tilde{F}_{c_{B1}} &= f(1 - c_{B1}) - \frac{f}{Xeq_1} x_B c_{B1}
\end{aligned}
$$

$$
\begin{aligned}
\tilde{D}_{x_A} &= c_{A0}c_{A1}g_{00} + c_{A0}(1-c_{A1})g_{01} \\
&\quad +(1-c_{A0})c_{A1}g_{10} + (1-c_{A0})(1-c_{A1})g_{11} + k \cdot x_A \\
\tilde{D}_{x_B} &= c_{B0}c_{B1}g_{00} + c_{B0}(1-c_{B1})g_{01} \\
&\quad +(1-c_{B0})c_{B1}g_{10} + (1-c_{B0})(1-c_{B1})g_{11} + k \cdot x_B \\
\tilde{D}_{c_{A0}} &= f(1-c_{A0}) + \frac{f}{Xeq_1}x_B c_{A0} \\
\tilde{D}_{c_{A1}} &= f(1-c_{A1}) + \frac{f}{Xeq_2}x_A c_{A1} \\
\tilde{D}_{c_{B0}} &= f(1-c_{B0}) + \frac{f}{Xeq_1}x_A c_{B0} \\
\tilde{D}_{c_{B1}} &= f(1-c_{B1}) + \frac{f}{Xeq_1}x_B c_{B1}
\end{aligned}
$$

The steady state probability distribution provides a global landscape picture. Compared with coupled 16 discrete landscapes, a global unified landscape with continuous variables in the extended dimensions captures the non-equilibrium property of the system. Non zero flux emerges in extended space as a measure of the system being at non-equilibrium and irreversible. It also provides a clear picture not only for a unified landscape but also for dynamics covering both intra continuous and inter discrite landscapes dynamics. We can see from this formalism that deterministic force and intrinsic fluctuation lie not only within each discrete landscape in x-space, but also in the hopping processes that are quantified by continuous c-variables. The dynamics in extended space can influence the attractors on the landscape and fluctuation induced transitions.

In particular, the optimal transition path can be quantified under this unified framework in continuous extended space. Fig. 5(b) shows the optimal path obtained using minimal action approach under 6-dimensional unified landscape projected to 2-dimensional $n_A - n_B$ space. Compared with optimal path at adiabatic limit, it's more tilted towards the 'off-off' state. This is due to the location change of intermediate state as a result of the weakened

coupling, the change of gradient force of the landscape and non-equilibrium flux.

Stability and Heterogeinity

As we can see from Fig.2 in main text, the landscape at adiabatic limit, extreme non-adiabatic limit and moderate non-adiabatic regime are very different. In the extreme non-adiabatic region, $\mathbf{H}_b$ terms are much smaller than $\mathbf{H}_0$ terms. The gene states defined by $\mathbf{H_0}$ are decoupled and we can expect as many discrete states as the dimension of $\mathbf{H_0}$ matrix. In our examples, it is up to 16 states. In the other limit where $\mathbf{H}_b$ dominates over $\mathbf{H_0}$, adiabatic approximation is valid and the system is mapped to an N-dimensional network whose number of stable states tends to be less. The moderate non-adiabatic regime, as we can see, is different from either limit. The location of the stable states are close to the ones in the extreme non-adiabatic regime, but the smaller basins that are close to larger basins merge to major peaks which is similar to the case in adiabatic limit.

To measure the heterogeneity, we take a look at Fano factor. Fano factor is defined as *variance/mean* and it measures the degrees of fluctuations. It is one if the process is purely random and the distribution is Poisson. Large Fano factor indicates large deviation from Poisson distribution and large fluctuation. As Fig 3 shows, Fano factor increases as adiabaticity decreases when ω is large and doesn't change much when ω is small. This is to be expected. When ω is large the hopping processes reach equilibrium, the 16 discrete states are strongly coupled. We are left with less steady states. Fano factor decreases as ω increases in this regime. However when ω is small enough and all the discrete state peaks are decoupled, the topology of the landscape won't change and Fano factor won't change significantly. It will even decrease a little bit as ω decreases due to less fluctuations in hopping processes.

The fact that at non-adiabatic regime there are more steady states and the steady states are more fluctuating all suggest there are more phenotype states at non-adiabatic regime.

Mean First Passage Time (MFPT) is the average of first passage time. It measures

the average time a successful transition takes and the transition rate is often defined as $r = 1/MFPT$. Fig. 7 shows how MFPT from normal state to cancer state changes with respect to adiabaticity. It suggests transitions at adiabatic limit and extreme non-adiabatic limit are rare and there exists an optimal transition rate at moderate ω non-adiabatic region. MFPT becomes larger at small ω non-adiabatic region, since the 16 discrete landscapes are decoupled and the system tends to stay in one of the landscape for a long time. It takes several jumps from the initial state to the final state. In our example from the initial 0110 state to the final 1001 state (1 stands for bound state and 0 unbound state for each of the four binding sites), it takes at least 4 jumps and the chance of this happening is relatively rare. At adiabatic limit the hopping processes occur so frequently that the genes won't stay in one of the discrete states for a long time. There is no sufficient time for protein copy numbers to reach the transcription level corresponding to the gene state. The moderate ω non-adiabatic regime, benefits from both proper jumping probability as well as sufficient residence time for transcription and as a result has the larger transition rate.

Fig. 5 in main text shows the optimal path under adiabatic limit using adiabatic approximation and moderate ω non-adiabatic regime using unified landscape approximation. The two optimal paths deviate from each other. There's no doubt that the optimal path is the transition path with maximum possibility among all the transition paths, but will optimal path give major contribution to total transition rate? Probably not in all circumstances. We can answer this question by checking the distribution of FPT. As Fig. 4 shows, the FPT distributions at both large ω and small ω region are relatively narrow around MFPT with little tails at larger FPT side. The moderate ω non-adiabatic regime surprisingly has a large diversity in FPT distribution. This suggests that in both large and small ω limit, most transition paths follow optimal path, resulting in the sharp concentration in FPT. But in moderate ω non-adiabatic regime, due to the relatively easy transition of gene states and sufficient transcription time between gene state changes, the transition paths are rather diversified, resulting in the broad FPT distribution. In this regime, starting from the initial

normal state, the cell is likely to travel to several different intermediate states before it finally reaches cancer state.

All the analysis we performed is based on the dynamic master equation Eq. (1) and fluctuations are intrinsic. The transitions between different phenotype states are induced by intrinsic noise. In non-adiabatic regime, more intermediate states show up in addition to the normal state and cancer state corresponding to more possible phenotype diversifications. Larger Fano factor and smaller MFPT indicates steady states are less stable and transitions are easier to occur. The transition paths, at adiabatic limit are well concentrated around optimal path which goes through the intermediate state while the optimal paths in moderate ω non-adiabatic regime are rather diversified indicating more intermediate states will be visited with more phenotype alternations.

All these results suggest moderate ω non-adiabatic regime to have larger heterogeneity. But where does the heterogeneity come from? The number of phenotype states decreasing as ω increasing is relatively easy to explain. The total of 16 states at small ω is the maximum number of discrete states the system can have. As ω increases, the coupling between the 16 states strengthens and the smaller basins on the landscape merge into larger basins. As a result, we have less number of states. The optimal transition rate and diversification of transition paths at moderate ω non-adiabatic regime is harder to understand. The unified landscape in extended space picture shed light on this issue. From the 6 dimensional dynamics in extended space we can see we need to consider deterministic dynamics and fluctuations of both protein concentration in x-variables and binding/unbinding processes in c-variables. At adiabatic limit, the system is close to steady state of $\mathcal{H}_b$. Gene states change so frequently that there is not enough time for protein concentration to reach transcription level of the final state. Transitions in x-space are difficult to occur. At small ω non-adiabatic limit, the system is close to steady state of $\mathcal{H}_0$. System tends to stay in one of the 16 steady states of $\mathcal{H}_0$ and transitions in c-space (change of gene states) are difficult to occur. It is only in moderate ω non-adiabatic regime where $\mathcal{H}_0$ rates and $\mathcal{H}_b$ rates are comparable, transitions can

occur in both x-space and c-space. In this regime transition rate is optimal and transition paths are diversified.

References

(1) Gillespie, D. T. Exact stochastic simulation of coupled chemical reactions. *The journal of physical chemistry* **1977**, *81*, 2340–2361.

(2) Kepler, T. B.; Elston, T. C. Stochasticity in transcriptional regulation: origins, consequences, and mathematical representations. *Biophysical journal* **2001**, *81*, 3116–3136.

(3) Schultz, D.; Onuchic, J. N.; Wolynes, P. G. Understanding stochastic simulations of the smallest genetic networks. *The Journal of chemical physics* **2007**, *126*, 245102.

(4) Wang, J.; Xu, L.; Wang, E. Potential landscape and flux framework of nonequilibrium networks: Robustness, dissipation, and coherence of biochemical oscillations. *Proceedings of the National Academy of Sciences* **2008**, *105*, 12271–12276.

(5) Wang, J.; Xu, L.; Wang, E.; Huang, S. The potential landscape of genetic circuits imposes the arrow of time in stem cell differentiation. *Biophysical journal* **2010**, *99*, 29–39.

(6) Wang, J.; Zhang, K.; Wang, E. Kinetic paths, time scale, and underlying landscapes: A path integral framework to study global natures of nonequilibrium systems and networks. *The Journal of chemical physics* **2010**, *133*, 125103.

(7) Feng, H.; Zhang, K.; Wang, J. Non-equilibrium transition state rate theory. *Chemical Science* **2014**, *5*, 3761–3769.

(8) Feynman, R. P.; Hibbs, A. R. *Quantum mechanics and path integrals*; McGraw-Hill New York, 1965; Vol. 2.

(9) Olender, R.; Elber, R. Calculation of classical trajectories with a very large time step: Formalism and numerical examples. *The Journal of chemical physics* **1996**, *105*, 9299–9315.

(10) Faccioli, P.; Sega, M.; Pederiva, F.; Orland, H. Dominant Pathways in Protein Folding. *Phys. Rev. Lett.* **2006**, *97*, 108101.

(11) Sasai, M.; Wolynes, P. G. Stochastic gene expression as a many-body problem. *Proceedings of the National Academy of Sciences* **2003**, *100*, 2374–2379.

(12) Zhang, K.; Sasai, M.; Wang, J. Eddy current and coupled landscapes for nonadiabatic and nonequilibrium complex system dynamics. *Proceedings of the National Academy of Sciences* **2013**, *110*, 14930–14935.

(13) Zhang, B.; Wolynes, P. G. Stem cell differentiation as a many-body problem. *Proceedings of the National Academy of Sciences* **2014**, *111*, 10185–10190.
